# A Coagulation Defect in Amyloidosis

**Published:** 1961-07

**Authors:** I. D. Fraser, M. R. Wills

**Affiliations:** Department of Pathology, United Bristol Hospitals; Department of Pathology, United Bristol Hospitals


### A COAGULATION DEFECT IN AMYLOIDOSIS

BY

I. D. FRASER AND M. R. WILLS

*Department of Pathology, United Bristol Hospitals*

The association of the presence of an abnormal serum protein fraction between the $\beta$ and $\gamma$ globulins in reticulo-endothelial disease and lymphatic leukaemia is a well recognized phenomenon (Rundles *et al.*, 1954; Buffa and Rappaport, 1957; Owen *et al.*, 1959). The combination of leukaemia and amyloidosis is also recognized, but the presence of a coagulation defect in amyloidosis must be relatively uncommon.

### CASE HISTORY

A man aged 60 was first seen in another hospital in October 1958 with a four week history of pain in the left hypochondrium and a feeling of fulness in the lower abdomen after meals. He had recently noted a swelling in the left upper quadrant. On examination, the liver and the spleen were both enlarged, and lymph glands were palpable in both axillae.

*Investigations:* The chest radiograph was within normal limits; barium meal examination showed a healed duodenal ulcer. Haemoglobin 10·4 g per 100 ml., total white cell count 14,000 per cmm. (neutrophils 10 per cent, eosinophils 1 per cent, monocytes 4 per cent, lymphocytes 85 per cent). The film appearances of the red cells and platelets was normal, the majority of the lymphocytes appeared primitive in morphology.

*November* 1958*:* Total white cell count and film appearance remained unchanged. *Sternal marrow:* the only abnormality noted was that 35 per cent of the total nucleated cell count were lymphocytes, 6·5 per cent being lymphoblasts. The M.E. ratio was 1·8 :1·0. A diagnosis of lymphatic leukaemia was made.

*January* 1959*:* He was admitted to this hospital for 3 days and given 5 millicuries of $P_{32}$. One month after treatment the haemoglobin was 12·1 g. per 100 ml. and the total white cells count 8,700 per cmm. of which 87 per cent were mature lymphocytes. The axillary glands had receded in size, and the spleen and liver were only just palpable. He remained reasonably well until June when he developed purpura over both legs; haemoglobin 8·6 g. per 100 ml., total white cell count 10,000 per cmm., of which 74 per cent were mature lymphocytes; platelets 300,000 per cmm.

In September he still had purpura over both legs and as he had developed painful knee joints, he was started on a course of prednisolone 10 mg. t.d.s. Within a month he was free from joint pain and the purpura had disappeared. In December he was readmitted to another hospital as there was a recurrence of purpura and bruising. Haemoglobin 7·4 g. per 100 ml., total white cell count 18,000 per cmm. (lymphocytes 29 per cent), platelets 450,000 per cmm. The white cells appeared normal but scanty normoblasts were noted. Total protein 7·0 g. per 100 ml., serum electrophoresis showed the presence of an abnormal fraction between the $\beta$ and $\gamma$ globulins. Radiographs of the skull, pelvis, humeri and femora were normal.

*March* 1960. In view of deterioration in his condition and a recurrence of hepato-splenomegaly, together with generalized bruising and purpura, he was transferred to this hospital. On examination, the liver and spleen were enlarged three fingers breadths below the right and left costal margins, having smooth firm edges. There was no evidence of congestive heart failure or oedema. Haemoglobin 7·1 g. per 100 ml., total white cell count 9,000 per cmm. (lymphocytes 45 per cent), platelets 200,000 per cmm. The white cells appeared normal but scanty normoblasts were seen on film examination.

Bleeding and clotting times were within normal range, and clot retraction was normal. A one-stage prothrombin time was carried out, the patient's time being 28 seconds and a normal control 15 seconds. The prothrombin time could be corrected to 18 seconds by the addition of one tenth volume of normal serum, but not by alumina plasma; the defect was therefore in a "serum" factor. This was confirmed in the thromboplastin generation test. Thromboplastic generation was defective when the patient's serum was incubated with normal alumina plasma and inosithin. The serum defect was corrected by using equal volumes of the patient's serum and a normal serum, and it was also corrected by equal volumes of the patients' serum and the serum from a known case of Christmas disease. (See Table).

TABLE I

| Source of serum | Substrate plasma clotting times at intervals of: | | | | | |
|---|---|---|---|---|---|---|
| | 2 mins. | 3 mins. | 4 mins. | 5 mins. | 6 mins. | 7 mins. |
| Normal | 21″ | 11″ | 9″ | 9″ | 8·5″ | 8·5″ |
| Patient | 49″ | 28″ | 25″ | 22″ | 21″ | 22″ |
| Christmas disease + Patient } equal volumes | 47″ | 17″ | 13″ | 12″ | 11″ | 11″ |
| Christmas disease + Normal } equal volumes | 43″ | 18″ | 13″ | 11·5″ | 11″ | 11″ |

*Thromboplastin generation* The generating system consisted of inositol phosphate as platelet substitute, normal alumina plasma, and the various sera as indicated.

Total protein 6·1 g. per 100 ml. (albumin 2·30, $\alpha_1$ 0·40, $\alpha_2$ 0·75, $\beta+\gamma$ 2·65); on electrophoresis an abnormal protein band of "myeloma" type was present between the $\beta$ and $\gamma$ globulins. Proteinuria was present but no Bence Jones protein was detected by routine methods and urinary electrophoresis showed no abnormal protein. Stool occult blood strongly positive.

His condition continued to deteriorate and on 3rd April he developed sudden severe pain in the left iliac fossa, the pulse rate rose to 140 per minute and the temperature was 101°F. On examination his abdomen was rigid. Laparotomy was performed but no perforated viscus was found, the only abnormality being considerable mesenteric haemorrhage. He developed post-operative bronchopneumonia and died six days later despite chemotherapy.

POST MORTEM EXAMINATION

The prominent findings were that the spleen and liver were enlarged. The spleen (950 g.) was firm and the cut surface showed the typical appearance of amyloidosis. Histologically the splenic pattern was completely obliterated by amyloid deposition. The cut surface of the liver (1950 g.) also showed typical amyloid changes; microscopically there was widespread deposition of amyloid substance, especially around the sinusoids, and almost every cell was damaged. Amyloid was also demonstrated in the kidney, stomach and intestines, and scanty deposits were noted in the heart and skeletal muscle.

There was no evidence of any chronic sepsis, tuberculosis, or glandular enlargement.

DISCUSSION

The initial enlargent of the liver and spleen could be explained by leukaemic infiltrations as these organs diminished in size after treatment with radio-active phosphorus. The return of the hepato-splenomegaly seems likely to be associated with the onset of amyloidosis. The combination of amyloidosis and leukaemia was mentioned by Bero (1957). Amyloidosis may also be associated with diseases of the reticulo-endothelial system including Hodgkin's diease and myelomatosis. Leonard (1957) described a patient with chronic lymphatic leukaemia who developed the nephrotic syndrome, the latter occurring as a result of amyloidosis. Teilum (1954) and Spain (1956) noted rapid development of amyloidosis in Hodgkin's disease when nitrogen mustard was used therapeutically. We know of no association between amyloidosis and $P_{32}$ treatment.

As there was no clinical or radiological evidence to support a diagnosis of myelomatosis, the abnormal serum protein of the "myeloma" type in this case would seem to be related to lymphatic leukaemia (Rundles *et al.*, 1954; Buffa and Rappaport, 1957; Owen *et al.*, 1959), although an abnormal protein fraction has been described in primary amyloidosis (Block *et al.*, 1956).

An interesting feature was the absence of purpura or bruising in the presence of a big liver and spleen when the patient was first seen. After $P_{32}$ treatment, and the subsequent re-enlargement of these organs, purpura and bruising developed. The platelets were normal but the thromboplastin generation test showed an apparent Christmas factor defect, the latter was corrected with serum from a known case of Christmas disease. A similar hereditary coagulation defect (Stuart clotting factor) was described by Houghie, Barrow and Graham (1957).

A consideration of the results of the coagulation studies detailed above restricts the patient's haemostatic deficiency to a lack of either Stuart factor or factor VII, or both. Uncomplicated factor VII deficiency (as a congenital state) does not affect the activity of serum in the thromboplastin generation test, and therefore the serum defect we observed must be ascribed to a deficiency of Stuart factor. However we cannot be sure that some factor VII deficiency was not also present. The use of "Stypven" in the one-stage prothrombin test could not be expected to reveal any minor defect of factor VII, since this reagent corrects the abnormal one-stage prothrombin time not only in factor VII deficiency but also in some examples of the Stuart defect (Telfer *et al.*, 1956).

Rabiner and Kretchmer (1961) demonstrated that the concentration of Stuart factor activity was in the $\alpha$- and $\beta$-globulin fractions of normal serum. The $\alpha_1$ and $\alpha_2$ globulin fractions were normal in our patient but, owing to the presence of a "myeloma" type band, we are unable to assess whether there was any deficiency of the $\beta$-globulin fraction, to account for the Stuart factor defect.

The coagulation defect could be ascribed to liver cell damage as the liver was heavily involved with amyloid substance. Chambers, Medd and Spencer (1958) found purpura in one out of six cases of primary amyloidosis and suggested the mechanism was "probably due to rupture of thickened, inelastic vessels"; no coagulation studies were done in this case. Symmers (1956) described two patients with primary amyloidosis who had unexplained haemorrhagic phenomena; in one case the liver was fairly heavily involved with amyloid but only mildly so in the other. In view of the mild liver damage in one of Symmer's cases, the possibility exists that in some obscure way it is the presence of amyloid substance that interferes with coagulation.

### SUMMARY

A patient with lymphatic leukaemia is described who after $P_{32}$ treatment developed amyloidosis with haemorrhagic manifestations. The latter were due to a serum defect in coagulation. It is tentatively concluded that the coagulation defect was an acquired Stuart factor defect.

Our thanks are due to Dr. A. L. Taylor for permission to publish the post motem findings, to Dr. R. C. Tudway for allowing us access to the notes, to Mr. R. J. Cousins, F.I.M.L.T. for technical assistance, and to Dr. A. B. Raper for helpful criticism.

### REFERENCES

Bero, G. L. (1957). Ann. intern. Med., **46**, 931.
Block, W. D., Rukavina, J. G. and Curtis, A. C. (1956). *J. Lab. clin. Med.*, **47**, 357.
Buffa, F., Rappaport, H. (1957). *Amer. J. Med.*, **22**, 504.
Chambers, R. A., Medd, W. E. and Spencer, H. (1958). *Quart. J. Med.*, **51**, 207.
Goldstein, R and Alexander, B. (1957). *Haemophilia and Haemophiloid Diseases*, p. 98. Edited K. M. Brinkhouse. University of North Carolina Press.
Houghie, C., Barrow, E. M. and Graham, J. B. (1957). *J. clin. Invest.*, **36**, 485.
Leonard, B. J. (1957). *Lancet*, **i**, 1356.

Owen, J. A., Pitney, W. R. and O'Dea, J. F. (1959). *J. clin. Path.*, **12**, 344.
Rabiner, S. F. and Kretchmer, N. (1961). *Brit. J. Haemat.*, **7**, 99.
Rundles, R. W., Conrad, E. V. and Arends, T. (1954). *Amer. J. Med.*, **16**, 842.
Spain, D. M. (1956). *Amer. J. clin. Path.*, **26**, 52.
Symmers, W. St. C. (1956). *J. clin. Path.*, **9**, 212.
Teilum, G. (1954). *J. lab. clin. Med.*, **43**, 367.
Telfer, T. P., Denson, K. W. and Wright, D. R. (1956). *Brit. J. Haemat.*, **2**, 308.

